# Supplementary material for: Development and Validation of the Vietnamese Children’s Short Dietary Questionnaire to Evaluate Food Groups Intakes and Dietary Practices among 9–11-Year-Olds Children in Urban Vietnam
Source: Nutrients. 2022 Sep 26;14(19):3996. doi: 10.3390/nu14193996 (PMC9570706; doi:10.3390/nu14193996)
Supplement: Supplementary file 1 [file nutrients-14-03996-s001.zip › nutrients-1891840-supplementary.pdf]

Table S1. STROBE-nut: An extension of the STROBE statement for nutritional epidemiology

Lachat C et al. (2016) STrengthening the Reporting of OBservational studies in Epidemiology – Nutritional Epidemiology (STROBE-nut): an extension of the STROBE statement. Plos Medicine 13(6) http://dx.doi.org/10.1371/journal.pmed.1002036 pdf or online version.

Item	Item nr	STROBE recommendations	Extension for Nutritional Epidemiology studies (STROBE-nut)	Reported on page #
**Title and abstract**	1	(a) Indicate the study's design with a commonly used term in the title or the abstract.<br><br>(b) Provide in the abstract an informative and balanced summary of what was done and what was found.	**nut-1** State the dietary/nutritional assessment method(s) used in the title, abstract, or keywords.	Page 1
**Introduction**				
Background rationale	2	Explain the scientific background and rationale for the investigation being reported.		Page 1-2
Objectives	3	State specific objectives, including any pre-specified hypotheses.		Page 2
**Methods**				
Study design	4	Present key elements of study design early in the paper.		Page 2
Settings	5	Describe the setting, locations, and relevant dates, including periods of recruitment, exposure, follow-up, and data collection.	**nut-5** Describe any characteristics of the study settings that might affect the dietary intake or	Page 3

Item	Item nr	STROBE recommendations	Extension for Nutritional Epidemiology studies (STROBE-nut)	Reported on page #
			nutritional status of the participants, if applicable.	
Participants	6	a) Cohort study—Give the eligibility criteria, and the sources and methods of selection of participants. Describe methods of follow-up.<br><br>Case-control study—Give the eligibility criteria, and the sources and methods of case ascertainment and control selection. Give the rationale for the choice of cases and controls.<br><br>Cross-sectional study—Give the eligibility criteria, and the sources and methods of selection of participants.<br><br>(b) Cohort study—For matched studies, give matching criteria and number of exposed and unexposed.<br><br>Case-control study—For matched studies, give matching criteria and the number of controls per case.	**nut-6** Report particular dietary, physiological or nutritional characteristics that were considered when selecting the target population.	Page 3
Variables	7	Clearly define all outcomes, exposures, predictors, potential confounders, and effect modifiers. Give diagnostic criteria, if applicable.	**nut-7.1** Clearly define foods, food groups, nutrients, or other food components.	Page 3-8

Item	Item nr	STROBE recommendations	Extension for Nutritional Epidemiology studies (STROBE-nut)	Reported on page #
			**nut-7.2** When using dietary patterns or indices, describe the methods to obtain them and their nutritional properties.	
Data sources - measurements	8	For each variable of interest, give sources of data and details of methods of assessment (measurement).Describe comparability of assessment methods if there is more than one group.	**nut-8.1** Describe the dietary assessment method(s), e.g., portion size estimation, number of days and items recorded, how it was developed and administered, and how quality was assured. Report if and how supplement intake was assessed.	Page 4-6
			**nut-8.2** Describe and justify food composition data used. Explain the procedure to match food composition with consumption data. Describe the use of conversion factors, if applicable.	Page 6-7
			**nut-8.3** Describe the nutrient requirements, recommendations, or dietary guidelines and the evaluation approach used to compare intake with the dietary reference values, if applicable.	NA
			**nut-8.4** When using nutritional biomarkers, additionally use the STROBE Extension for Molecular Epidemiology (STROBE-ME). Report the type of biomarkers used and their usefulness as dietary exposure markers.	NA

Item	Item nr	STROBE recommendations	Extension for Nutritional Epidemiology studies (STROBE-nut)	Reported on page #
			**nut-8.5** Describe the assessment of nondietary data (e.g., nutritional status and influencing factors) and timing of the assessment of these variables in relation to dietary assessment.	Page 6-7
			**nut-8.6** Report on the validity of the dietary or nutritional assessment methods and any internal or external validation used in the study, if applicable.	NA
Bias	9	Describe any efforts to address potential sources of bias.	**nut-9** Report how bias in dietary or nutritional assessment was addressed, e.g., misreporting, changes in habits as a result of being measured, or data imputation from other sources	Page 7
Study Size	10	Explain how the study size was arrived at.		Page 3
Quantitative variables	11	Explain how quantitative variables were handled in the analyses. If applicable, describe which groupings were chosen and why.	**nut-11** Explain categorization of dietary/nutritional data (e.g., use of N-tiles and handling of nonconsumers) and the choice of reference category, if applicable.	Page 5-6
Statistical Methods	12	(a) Describe all statistical methods, including those used to control for confounding	**nut-12.1** Describe any statistical method used to combine dietary or nutritional data, if applicable.	NA

Item	Item nr	STROBE recommendations	Extension for Nutritional Epidemiology studies (STROBE-nut)	Reported on page #
		(b) Describe any methods used to examine subgroups and interactions.<br><br>(c) Explain how missing data were addressed.<br><br>(d) Cohort study—If applicable, explain how loss to follow-up was addressed.<br><br>Case-control study—If applicable, explain how matching of cases and controls was addressed.<br><br>Cross-sectional study—If applicable, describe analytical methods taking account of sampling strategy.<br><br>(e) Describe any sensitivity analyses.	**nut-12.2** Describe and justify the method for energy adjustments, intake modeling, and use of weighting factors, if applicable.<br><br>**nut-12.3** Report any adjustments for measurement error, i.e,. from a validity or calibration study.	NA<br><br>Page 7-9
**Results**				
Participants	13	(a) Report the numbers of individuals at each stage of the study—e.g., numbers potentially eligible, examined for eligibility, confirmed eligible, included in the study, completing follow-up, and analyzed.<br><br>(b) Give reasons for non-participation at each stage.<br><br>(c) Consider use of a flow diagram.	**nut-13** Report the number of individuals excluded based on missing, incomplete or implausible dietary/nutritional data.	Page 9

Item	Item nr	STROBE recommendations	Extension for Nutritional Epidemiology studies (STROBE-nut)	Reported on page #
Descriptive data	14	(a) Give characteristics of study participants (e.g., demographic, clinical, social) and information on exposures and potential confounders<br><br>(b) Indicate the number of participants with missing data for each variable of interest<br><br>© Cohort study—Summarize follow-up time (e.g., average and total amount)	**nut-14** Give the distribution of participant characteristics across the exposure variables if applicable. Specify if food consumption of total population or consumers only were used to obtain results.	Page 9-10
Outcome data	15	Cohort study—Report numbers of outcome events or summary measures over time.<br><br>Case-control study—Report numbers in each exposure category, or summary measures of exposure.<br><br>Cross-sectional study—Report numbers of outcome events or summary measures.		Page 9-15
Main results	16	(a) Give unadjusted estimates and, if applicable, confounder-adjusted estimates and their precision (e.g., 95% confidence interval).<br><br>Make clear which confounders were adjusted for and why they were included.	**nut-16** Specify if nutrient intakes are reported with or without inclusion of dietary supplement intake, if applicable.	NA

Item	Item nr	STROBE recommendations	Extension for Nutritional Epidemiology studies (STROBE-nut)	Reported on page #
		(b) Report category boundaries when continuous variables were categorized.<br>(c) If relevant, consider translating estimates of relative risk into absolute risk for a meaningful time period.		
Other analyses	17	Report other analyses done—e.g., analyses of subgroups and interactions and sensitivity analyses.	**nut-17** Report any sensitivity analysis (e.g., exclusion of misreporters or outliers) and data imputation, if applicable.	Page 9-10
**Discussion**				
Key results	18	Summarize key results with reference to study objectives.		Page 16
Limitation	19	Discuss limitations of the study, taking into account sources of potential bias or imprecision. Discuss both direction and magnitude of any potential bias.	**nut-19** Describe the main limitations of the data sources and assessment methods used and implications for the interpretation of the findings.	Page 19-20
Interpretation	20	Give a cautious overall interpretation of results considering objectives, limitations, multiplicity of analyses, results from similar studies, and other relevant evidence.	**nut-20** Report the nutritional relevance of the findings, given the complexity of diet or nutrition as an exposure.	Page 16-19
Generalizability	21	Discuss the generalizability (external validity) of the study results.		**Page 19**
**Other information**				

Item	Item nr	STROBE recommendations	Extension for Nutritional Epidemiology studies (STROBE-nut)	Reported on page #
Funding	22	Give the source of funding and the role of the funders for the present study and, if applicable, for the original study on which the present article is based.		Page 21
Ethics			**nut-22.1** Describe the procedure for consent and study approval from ethics committee(s).	Page 3&9
Supplementary material			**nut-22.2** Provide data collection tools and data as online material or explain how they can be accessed.	Page 21

Figure S1: Vietnamese Children' Short Dietary Questionnaire (downloaded from Key Survey)

P2_Câu hỏi ngắn về chế độ ăn của học sinh 9 - 11 tuổi

Bảng câu hỏi này nhằm mục đích tìm hiểu chế độ ăn và vận động của em trong tuần vừa qua (trong 7 ngày vừa qua tính từ thời điểm này). Các em hãy đọc các ví dụ sau đây để hiểu rõ cách trả lời câu hỏi:
Trong tuần qua, nếu em không uống sữa không đường hoặc ăn phô mai, sữa chua không đường, em click vào lựa chọn không ăn
Trong tuần qua, nếu em ăn 1 mẩu bánh mì, em click vào lựa chọn ít hơn 1 lần/ tuần.
Trong tuần qua, em ăn cơm mỗi ngày 2 lần vào buổi trưa và tối, và ăn sáng mỗi ngày các món ăn từ gạo (bún/ phở...), em click vào lựa chọn 2-3 lần/ngày.
Trong tuần qua, có 3 ngày em ăn trái cây, Nếu em ăn 1 lần mỗi ngày, lựa chọn của em là 2-4 lần/ tuần. Nếu em ăn 2 lần mỗi ngày, nghĩa là em ăn tổng cộng 3 ngày x 2 lần/ngày = 6 lần, em click vào lựa chọn 5-6 lần/tuần. Nếu em ăn 3 lần mỗi ngày, nghĩa là em ăn tổng cộng 3 ngày x 3 lần/ ngày = 9 lần, vì không có lựa chọn 9 lần/ngày nên em sẽ click vào lựa chọn 1 lần/ ngày
Nếu có nội dung nào chưa rõ, đừng ngại hỏi các cô/chú trong nhóm nghiên cứu để giải thích các thắc mắc của mình. Các nội dung em điền vào sẽ được giữ bí mật và chỉ sử dụng cho mục đích nghiên cứu.

Vui lòng chọn ngôn ngữ

○

○ Tiếng Việt

Phần 1. Thông tin cơ bản

Các em vui lòng điền mã số đã được gửi vào ô bên dưới

Trường

....

Lớp

....

Mã số được phát

....

Phần luyện tập

Câu hỏi 1: Ví dụ trong tuần vừa qua, ngày nào em cũng ăn 2 bữa cơm với thịt và 1 bữa cơm với tôm. Như vậy, trong tuần qua, em ăn "thịt các loại, trứng, cá, tôm" bao nhiêu lần? (Chọn 1 đáp án đúng nhất trong các lựa chọn sau)

	Không ăn	Ít hơn 1	1 lần/ tuần	[illegible] tuần	5-6 lần/	1 lần/	2-3 lần/	≥4 lần/
Thịt các loại (thịt heo, thịt bò, thịt gà), trứng.	○	○	○	○	○	○	○	○

Câu hỏi 2: Ví dụ trong tuần qua, từ thứ 2 đến thứ 6 em ăn trái cây 1 lần mỗi ngày, riêng thứ 7 em ăn trái cây 3 lần. Như vậy, trong tuần qua em trái cây bao nhiêu lần? (Em hãy chọn 1 đáp án đúng nhất trong các đáp án sau)

	Không ăn	Ít hơn 1	1 lần/ tuần	[illegible] tuần	5-6 lần/	1 lần/	2-3 lần/	≥4 lần/
Ăn trái	○	○	○	○	○	○	○	○

Phần 2. Câu hỏi ngắn về chế độ ăn

Trong tuần qua, em có ăn các loại thực phẩm sau đây không?								
	Không ăn	Ít hơn 1 lần/ tuần	1 lần/ tuần	2-4 lần/ tuần	5-6 lần/ tuần	1 lần/ ngày	2-3 lần/ ngày	≥4 lần/ ngày
Cơm hoặc các món như bún, phở, nui, hủ tiếu, bánh ướt	○	○	○	○	○	○	○	○
Bánh mì (bánh mì ổ, bánh mì sandwich, bánh mì que)	○	○	○	○	○	○	○	○
Khoai lang, khoai mì, khoai môn, khoai tây, bắp	○	○	○	○	○	○	○	○
Rau các loại	○	○	○	○	○	○	○	○
Trái cây	○	○	○	○	○	○	○	○
Nước ép trái cây nguyên chất (cam vắt không đường, ép táo, ép dưa hấu...)	○	○	○	○	○	○	○	○
Thịt (thịt bò, thịt heo, thịt gà, thịt vịt), cá, trứng	○	○	○	○	○	○	○	○
Các loại thịt chế biến (xúc xích, lạp xưởng, chả lụa, cá/bò viên, thịt xông khói, pa tê)	○	○	○	○	○	○	○	○
Đậu phụ, đậu hủ, đậu xanh	○	○	○	○	○	○	○	○
Các loại hạt (đậu phộng, hạt điều, óc chó, hạnh nhân…)	○	○	○	○	○	○	○	○

Trong tuần qua, em có ăn hoặc uống các loại thực phẩm sau đây không?								
	Không ăn	Ít hơn 1 lần/ tuần	1 lần/ tuần	2-4 lần/ tuần	5-6 lần/ tuần	1 lần/ ngày	2-3 lần/ ngày	≥4 lần/ ngày
Sữa không đường và các sản phẩm từ sữa bò không đường (sữa chua không đường, phô mai)	○	○	○	○	○	○	○	○
Sữa có đường và các sản phẩm từ sữa bò có đường (sữa chua có đường, sữa chua uống vị dâu/ cam..., sữa lúa mạch (milo),..)	○	○	○	○	○	○	○	○
Các loại thức uống có đường (nước ngọt có gas, trà sữa, nước tăng lực, nước uống thể thao, nước trái cây có đường, sữa đậu nành có đường,...)	○	○	○	○	○	○	○	○
Thức ăn vặt nhiều đường (bánh ngọt, bánh kem, bánh qui, bánh xốp kẹo, sô cô la, kem, chè...)	○	○	○	○	○	○	○	○
Thức ăn vặt nhiều muối (bánh snack, snack khoai tây, bánh tráng muối)	○	○	○	○	○	○	○	○
Thức ăn nhanh (pizza, gà rán, hamburger, khoai tây chiên, cá viên chiên...)	○	○	○	○	○	○	○	○
Ăn mì gói, mì ly (mì ăn liền)	○	○	○	○	○	○	○	○

Trong tuần qua, em có các thói quen sau đây không?							
	Chưa bao giờ	1 lần/tuần	2-4 lần/tuần	5-6 lần/tuần	1 lần/ngày	2-3 lần/ngày	≥ 4 lần/ngày
**Chấm thêm muối, nước mắm, nước tương trong khi ăn**	○	○	○	○	○	○	○
	○	○	○	○	○	○	○
**Thêm các loại nước sốt khác như tương cà, tương ớt, tương đen, mayonnaise, sốt BBQ...**	○	○	○	○	○	○	○
	○	○	○	○	○	○	○
**Xem ti vi, điện thoại, ipad trong khi ăn**	○	○	○	○	○	○	○

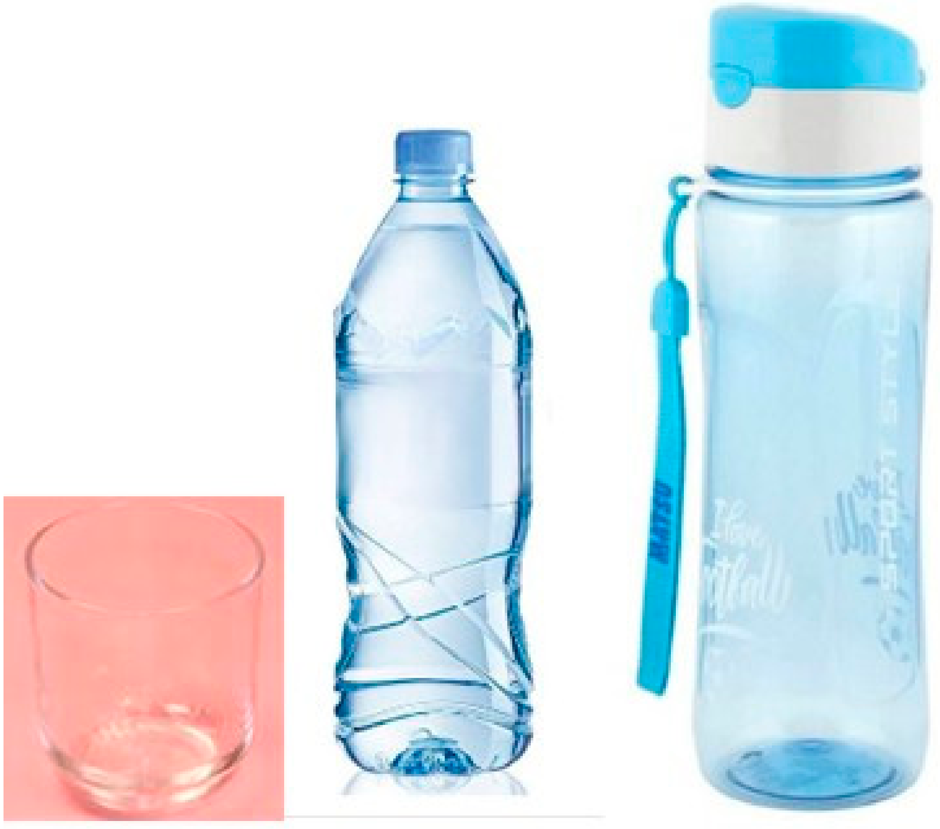

ly 200 ml chai 500ml chai 700ml

Trong tuần qua, trung bình mỗi ngày em uống bao nhiêu ly nước lọc/ nước đun sôi/ nước suối? (1 ly = 200 ml nước, 1 chai 500 ml =2,5 ly, 1 bình nước 700 ml = 3,5 ly)

- ○ Em không uống nước lọc Khoảng
- ○ 1 ly nước mỗi ngày
- ○
- ○ Khoảng 2- 3 ly nước hoặc 1 chai 500 ml mỗi ngày
- ○
- ○ Khoảng 4 - 5 ly nước hoặc 2 chai 500 ml mỗi ngày

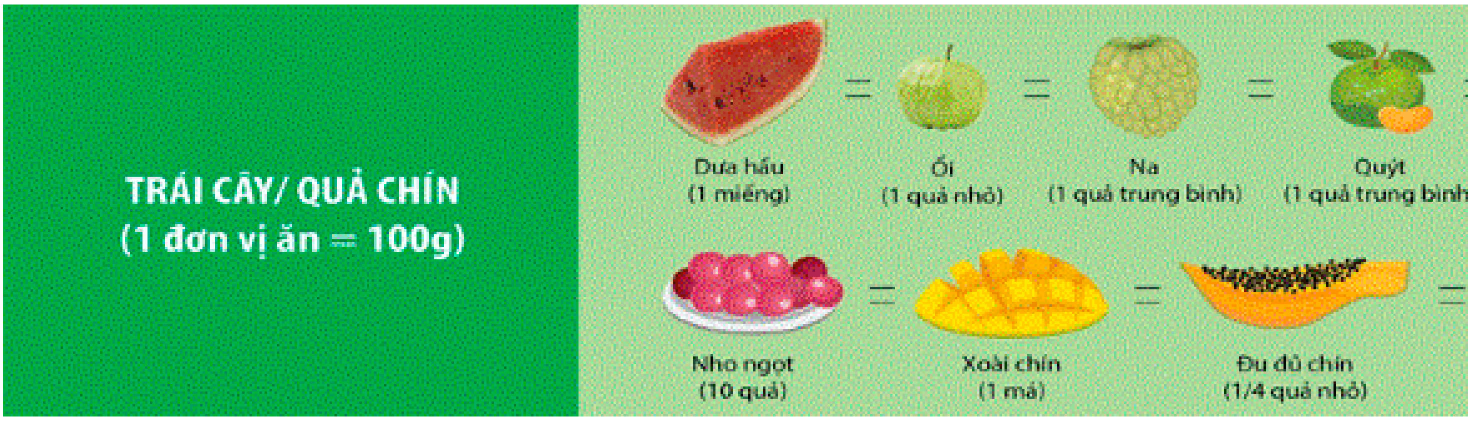

Trong tuần qua, trung bình mỗi ngày em thường ăn khoảng bao nhiêu phần trái cây, kể cả trái cây sấy khô và nước trái cây tươi? (1 phần tương đương với 1 đơn vị trái cây trong hình bên trên)

- ○ Em không ăn trái cây/ uống nước trái cây
- ○ Ít hơn 1 phần
- ○ 1 phần
- ○ 2 phần
- ○
- ○

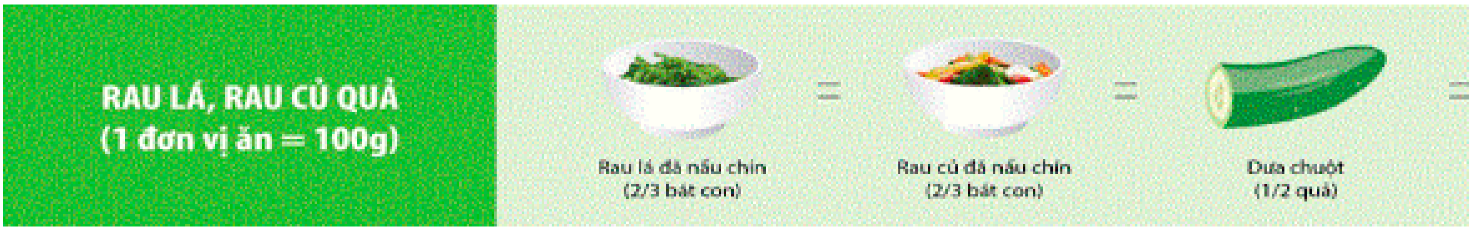

Trong tuần qua, trung bình mỗi ngày , em thường ăn bao nhiêu phần rau (gồm tất cả các loại rau trong canh, rau xào, rau luộc, gỏi, rau sống)? (1 phần rau tương đương với 1 đơn vị rau như hình bên trên)

- ○ Em không ăn rau
- ○ Ít hơn 1 phần
- ○ 1 phần
- ○ 2 phần
- ○
- ○

Trong tuần vừa qua, có bao nhiêu ngày em vận động hoặc tập thể dục làm cho em toát mồ hôi, hoặc tim đập nhanh hoặc thở khó với tổng thời gian ít nhất 1 tiếng/ngày? (Ví dụ: đi bộ nhanh, chạy nhảy, đá cầu, nhảy dây, đá bóng, ...)

- 1 ngày trong tuần qua hoặc ít hơn
- 2 ngày trong tuần qua
- 3 ngày trong tuần qua
- 4 ngày trong tuần qua
- 5 ngày trong tuần qua
- 6 ngày trong tuần qua

Thông thường, có bao nhiêu ngày trong tuần em vận động hoặc tập thể dục làm cho em toát mồ hôi, hoặc tim đập nhanh hoặc thở khó) với tổng thời gian ít nhất 1 tiếng/ngày? (Ví dụ: đi bộ nhanh, chạy nhảy, đá cầu, nhảy dây, đá bóng, ...)

- 1 ngày trong tuần hoặc íthơn
- 2 ngày trong tuần
- 3 ngày trong tuần
- 4 ngày trong tuần
- 5 ngày trong tuần
- 6 ngày trong tuần

Xem lại câu trả lời
